# Supplementary material for: Rapid sensing of l-leucine by human and murine hypothalamic neurons: Neurochemical and mechanistic insights
Source: Mol Metab. 2018 Feb 7;10:14–27. doi: 10.1016/j.molmet.2018.01.021 (PMC5985239; doi:10.1016/j.molmet.2018.01.021)
Supplement: Supplementary file 1 [file mmc1.docx]

**Supplementary Methods**

**Preparation of extraction and culture media**

Media were prepared as described in Supplementary Table 1 (without amino acids, glucose/pyruvate/lactate or B27), sterile filtered, stored at 4°C and used for up to 6 months. Osmolarity was adjusted to 260 mOsm using sucrose. The day before culturing cells, final extraction and culture media were prepared by adding B27 without Insulin (Life Technologies), Amino acid stock solutions (Supplementary Table 2) and glucose, pyruvate, and lactate (final concentrations: Glucose, 2.5 mM; Lactic Acid, 1 µM; Sodium Pyruvate, 0.23 mM). Papain (40 U/ml, PAP2, Worthington Biochemical) was prepared in extraction media (with amino acids, glucose, pyruvate and lactate, as above, but without calcium and B27) and diluted to a final concentration of 20 U/ml. Papain was not used for more than one week after reconstitution. aCSF was prepared as described in Supplementary Table 3), with amino acids added to the same concentration as in extraction and culture media (see Appendix 1), and 2.5 mM glucose.

Supplementary Table 1: Vitamin and Salt composition of culture and extraction media

| **Components** | **Culture media (mM)** | **Extraction media (mM)** |
| --- | --- | --- |
| **Vitamins** |  |  |
| Choline chloride | 0.029 | 0.029 |
| D-Calcium pantothenate | 0.008 | 0.008 |
| Folic Acid | 0.009 | 0.009 |
| Niacinamide | 0.033 | 0.033 |
| Pyridoxal hydrochloride | 0.020 | 0.020 |
| Riboflavin | 0.001 | 0.001 |
| Thiamine hydrochloride | 0.012 | 0.012 |
| Vita minutes B12 | 5.02E-06 | 5.02E-06 |
| i-Inositol | 0.040 | 0.040 |
| **Inorganic Salts** |  |  |
| Calcium Chloride | 1.801 | 1.801 |
| Ferric Nitrate | 2.48E-04 | 2.48E-04 |
| Magnesium Chloride | 0.814 | 0.814 |
| Potassium Chloride | 5.333 | 5.333 |
| Sodium Bicarbonate | 26.190 | 0.88 |
| Sodium Chloride | 51.724 | 76 |
| Sodium Phosphate monobasic | 0.906 | 0.906 |
| Zinc sulfate | 6.74E-04 | 6.74E-04 |
| **Other Components** |  |  |
| HEPES | 10.924 |  |
| Phenol Red | 0.022 | 0.0212 |
| MOPS |  | 10 |
|  |  |  |
| pH and Temperature | pH 7.6 at 37°C | pH 7.4 at 20°C |

Supplementary Table 2: Amino acid composition of extraction and culture media

| **Amino Acids** | **Final Concentration (mM)** |
| --- | --- |
| Glycine | 0.020 |
| L-Alanine | 0.036 |
| L-Arginine hydrochloride | 0.028 |
| L-Asparagine-H_2_O | 0.058 |
| L-Cysteine |  |
| L-Histidine HCl-H_2_O | 0.020 |
| L-Isoleucine | 0.003 |
| L-Leucine | 0.008 |
| L-Lysine hydrochloride | 0.067 |
| L-Methionine | 0.006 |
| L-Phenylalanine | 0.011 |
| L-Proline |  |
| L-Serine | 0.074 |
| L-Threonine | 0.055 |
| L-Tryptophan | 0.004 |
| L-Tyrosine | 0.009 |
| L-Valine | 0.004 |
| Glutamine | 0.100 |

Supplementary Table 3: aCSF salt composition (pH adjusted to 7.4).

|  | Concentration (mM) |
| --- | --- |
| NaCl | 138 |
| KCl | 4.5 |
| NaHCO_3_ | 4.2 |
| NaH_2_PO_4_ | 1.2 |
| HEPES | 10 |
| CaCl_2_ | 2.6 |
| MgCl_2_ | 1.2 |

**Supplementary Figure 1.**

**Supplementary Figure 1: Optimization of culture conditions.**

Effect of papain type (Worthington PAP vs. Worthington PAP2) on cell yield (A). Effect of media nutrient composition (Standard Hibernate A and Neurobasal A with high glucose and amino acid concentrations vs. in house Hibernate and Neurobasal with low glucose and amino acid composition, see Method section) on cell survival assessed by trypan blue staining (B). Effect of debris removal technique (4% BSA vs. layered 4% and 8% BSA) on debris in culture (C). Summary of optimized factors with an effect on cell yield, cell survival or debris in culture (d).
